# Reproducibility of a semiautomatic lobar lung tissue assignment technique on noncontrast CT scans: a study on swine animal model

**DOI:** 10.1186/s41747-024-00453-1

**Published:** 2024-05-06

**Authors:** Nile Luu, Nathan Van, Alireza Shojazadeh, Yixiao Zhao, Sabee Molloi

**Affiliations:** Department of Radiological Sciences, Medical Sciences I, B-140, University of California, Irvine, Irvine, CA 92697 USA

**Keywords:** Animals, Lung, Observer variation, Swine, Tomography (x-ray computed)

## Abstract

**Background:**

To evaluate the reproducibility of a vessel-specific minimum cost path (MCP) technique used for lobar segmentation on noncontrast computed tomography (CT).

**Methods:**

Sixteen Yorkshire swine (49.9 ± 4.7 kg, mean ± standard deviation) underwent a total of 46 noncontrast helical CT scans from November 2020 to May 2022 using a 320-slice scanner. A semiautomatic algorithm was employed by three readers to segment the lung tissue and pulmonary arterial tree. The centerline of the arterial tree was extracted and partitioned into six subtrees for lobar assignment. The MCP technique was implemented to assign lobar territories by assigning lung tissue voxels to the nearest arterial tree segment. MCP-derived lobar mass and volume were then compared between two acquisitions, using linear regression, root mean square error (RMSE), and paired sample *t*-tests. An interobserver and intraobserver analysis of the lobar measurements was also performed.

**Results:**

The average whole lung mass and volume was 663.7 ± 103.7 g and 1,444.22 ± 309.1 mL, respectively. The lobar mass measurements from the initial (MLobe1) and subsequent (MLobe2) acquisitions were correlated by MLobe1 = 0.99 MLobe2 + 1.76 (*r* = 0.99, *p* = 0.120, RMSE = 7.99 g). The lobar volume measurements from the initial (VLobe1) and subsequent (VLobe2) acquisitions were correlated by VLobe1 = 0.98VLobe2 + 2.66 (*r* = 0.99, *p* = 0.160, RSME = 15.26 mL).

**Conclusions:**

The lobar mass and volume measurements showed excellent reproducibility through a vessel-specific assignment technique. This technique may serve for automated lung lobar segmentation, facilitating clinical regional pulmonary analysis.

**Relevance statement:**

Assessment of lobar mass or volume in the lung lobes using noncontrast CT may allow for efficient region-specific treatment strategies for diseases such as pulmonary embolism and chronic thromboembolic pulmonary hypertension.

**Key points:**

• Lobar segmentation is essential for precise disease assessment and treatment planning.

• Current methods for segmentation using fissure lines are problematic.

• The minimum-cost-path technique here is proposed and a swine model showed excellent reproducibility for lobar mass measurements.

• Interobserver agreement was excellent, with intraclass correlation coefficients greater than 0.90.

**Graphical Abstract:**

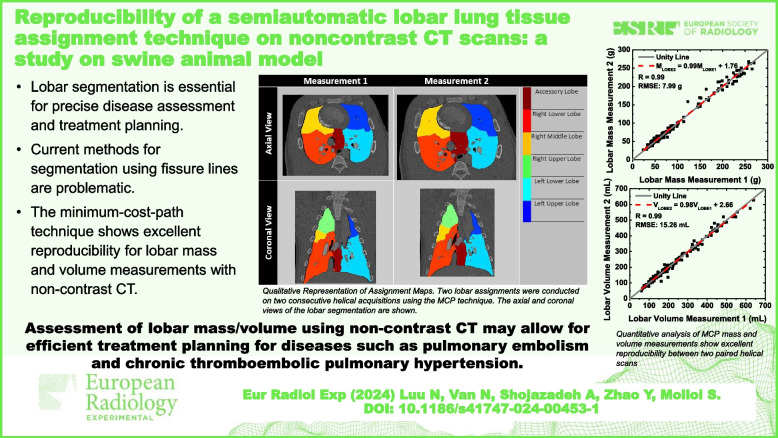

## Background

The automatic identification and segmentation of pulmonary lobes from medical imaging plays an important role in precise disease assessment and optimal treatment planning. Diseases such as pulmonary embolism or chronic thromboembolic pulmonary hypertension can benefit from lobar segmentation as it allows for the quantification of severity, facilitating risk stratification and providing prompt diagnostic strategies for different areas of the lung [1]. In addition, automatic lobar segmentation would be able to aid in disease assessment for emphysema and lung cancer to evaluate lung mass or volume changes [2, 3]. Thus, the assessment of individual lung lobes is necessary to provide effective treatment planning for patients in need.

Current methods for automatic lobar segmentation rely on lobar fissures and other anatomical based knowledge to provide an accurate regional assessment [4]. However, these fissures may appear incomplete or unclear on computed tomography (CT) scans of patients with pulmonary infiltrations or other lung abnormalities [5]. Similar issues arise for atlas-based [6, 7], airway-guided [8], and machine learning algorithms [9], as they depend on healthy lung anatomy and clear fissures for segmentation. Alternative methods that are used to quantify regional ventilation and perfusion scans usually are evaluated based on rectangularly shaped regions of interest that do not have any anatomical or physiological basis [10]. Hence, an approach that does not rely on fissures or anatomical knowledge may be an appropriate solution to these problems to create an accurate lobar segmentation of the lungs.

Recently, an automated minimum cost path (MCP) technique was utilized for accurate pulmonary lobar segmentation using CT pulmonary angiography images [11]. This method relies on the assumption that the lung tissue is supplied by the nearest arterial tree, facilitating the determination of the minimum distance between a lung tissue voxel and its closest supplying pulmonary artery [12]. The aim of this study was to assess the reproducibility of the MCP technique for lobar segmentation using noncontrast CT images in a swine model. The reproducibility of lobar segmentation was evaluated through the quantitative comparison of repeated lobar lung mass and volume measurements.

## Methods

### Animals

The study was approved by the Institutional Animal Care and Use Committee under the protocol AUP-18–191 at the University of California, Irvine, and was performed under specific guidelines for animal care. From November 2020 to May 2022, a total of 16 Yorkshire swine, weighing 49.9 ± 4.7 kg (mean ± standard deviation), were used to evaluate the reproducibility of the MCP technique on pulmonary CT scans. At least two subsequent acquisitions were performed on each swine to gather a reproducibility scan pair. For each swine, Telazol (4.4 mg/kg) and Xylazine (2.2 mg/kg) was intravenously injected for sedation. After endotracheal intubation, anesthesia was maintained over the course of the experiment via a mechanical ventilator with 1.5−2.5% isoflurane (Highland Medical Equipment, Temecula, CA and Baxter, Deerfield, IL, USA). Vital signs including oxygen saturation (%), heart rate (beats per min [bpm]), blood pressure (mmHg), and end-tidal carbon dioxide (mmHg) were monitored continuously. All animals were euthanized utilizing saturated potassium chloride (KCl) under deep anesthesia.

### CT protocol

A 320-row-detector CT clinical scanner (Aquilion One, Canon Medical Systems, Tustin, CA, USA) was used to acquire all CT examinations. Each swine was placed in the supine position and scanned in the head-to-toe direction. Tube voltage was set at 100 kVp, while tube current was set at 50 mA. The scan field of view was 320 mm, gantry rotation time 0.35 s, collimation 64 × 0.5 mm, with a pitch value of 1.48. For each swine, at least one pair of helical CT scans were obtained. A paired helical scan consisted of one noncontrast helical CT scan and a subsequent helical scan under the same conditions approximately 5 to 10 min later. Four of the sixteen animals had more than one pair of helical scans performed on them. This was done due to altered physiological states and potential fluid buildup. For these animals, delays between scan pairs varied from 20 min to 10 h, with reproducibility being assessed exclusively between scans from the same pair. A total of 46 scans were obtained. The CT dose index was also collected from the dose report sheet.

### MCP technique and image processing

**Fig. 1:**
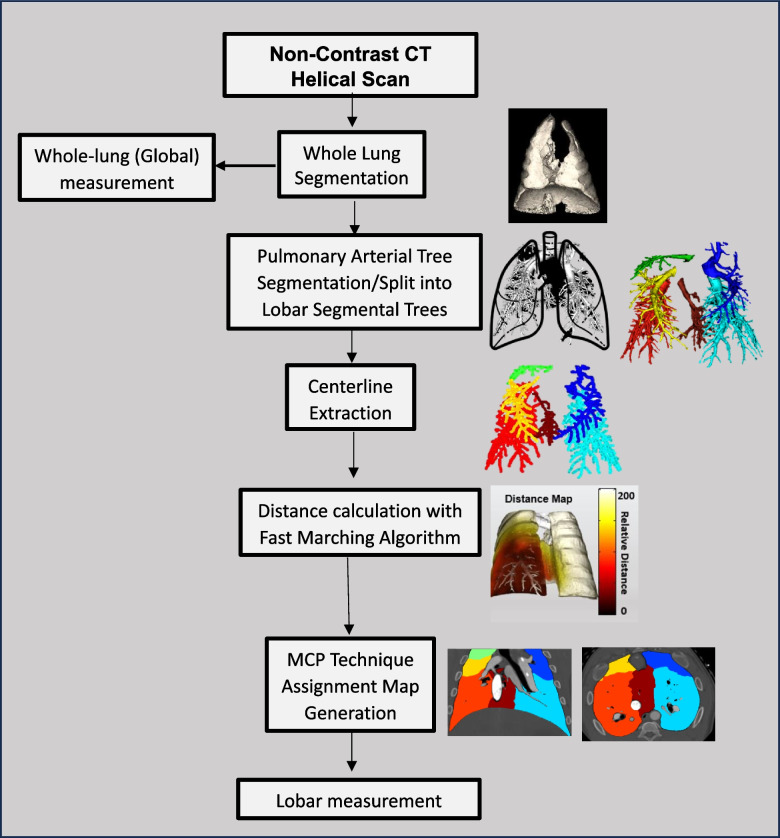
Study workflow. Flow chart describing the steps on how measurements were acquired. Initially, a whole lung segmentation was generated. The mass of the global lung was calculated using the whole lung segmentation. Next, a pulmonary arterial tree segmentation was semi-automatically extracted and was split into six different subtrees for the six lobes of the swine lung. Centerlines were extracted, distance calculations were performed automatically, and finally the minimum cost path (MCP) technique generated assignment maps, which were used to acquire the lobar measurements

The lung images from each of the CT acquisitions were registered using in-house MATLAB® software (version R2019a, MathWorks Inc, Natick, MA, USA). The entire lung parenchyma was then semiautomatically segmented using a Vitrea workstation (Vitrea fX version 7.14, Vital Images, Inc., Minnetonka, MN, USA) to generate a whole lung segmentation. Subsequently, the pulmonary arterial tree was segmented and divided into six distinct lobes of the swine lung: right upper lobe (RUL), right middle lobe (RML), right lower lobe (RLL), left upper lobe (LUL), left lower lobe (LLL), and the accessory lobe (AL), which is unique to the porcine lung [13]. Vessel centerlines were then extracted using in-house MATLAB software. These centerlines served as seed points, to calculate the distance to each specific lung voxel by employing a fast-marching algorithm. As a result of these calculations, distance maps were developed, assigning each voxel to its closest supplying artery. Consequently, lung voxels with a shorter distance to the centerline were assigned to the nearest lobe, while those farther away were assigned to neighboring lobar territories. Assignment maps were then generated and used to calculate the parenchymal mass for each lobe of the lung. The general workflow is shown in Fig. 1.

### Mass calculation

Following assignment, the non-air volumetric fraction of tissue (${T}_{f}$, %) was calculated from the noncontrast image with decomposition of the pure tissue (50 HU) and air (-1,000 HU). The HU value for each voxel was subtracted from the HU of air and divided by the difference between the HU for tissue and air (Eq. 1) [14]. The mass of each voxel was calculated to be the product of the voxel size (${Voxel}_{x,y,z}, {cm}^{3}$), the lung parenchymal tissue density (1.053 g/mL), and the non-air fraction of tissue (%) (Eq. 2) [14]. The tissue mass of the whole lung and each lobar territory was then estimated from the total amount of voxels in each territory.

1 $${T}_{f}=\frac{{HU}_{x,y,z}-{HU}_{Air}}{{HU}_{Tissue}-{HU}_{Air}}$$

2 $${M}_{x, y,z}={T}_{f}\times {Voxel}_{x,y,z}\times 1.053$$

### Volume calculation

The volume of the lungs was calculated in a global and lobar fashion for each CT acquisition. For this purpose, the voxel volume was determined from the voxel size (0.625 × 0.625 × 0.5 mm^3^). The total number of the voxels was then summed to measure the volume of each global and lobar parenchymal segmentation of the lung.

### Statistical analysis

Global and lobar mass measurements between paired acquisitions were compared using linear regression analysis, concordance correlation coefficients, paired *t*-tests, root mean square error (RMSE), Pearson correlation coefficient, and Bland–Altman plots. The mean ± standard deviation was used to present the average mass, volume, and average percent difference. The RMSE was also normalized to the mean. The *p* values lower than 0.05 were considered significant. In the Bland–Altman analysis, the mean difference was reported along with the 95% confidence interval (CI) (lower bound, upper bound). Excel® version 2307 (Microsoft, Redmond, WA, USA) was used for these calculations. The same statistical tests were performed for the volume data.

A blinded interobserver analysis was performed for all measurements between three independent observers, authors N.L., N.V., and A.Z., with 3, 1, and 2 years of medical imaging research experience respectively. This was performed as variability can be introduced during the semiautomatic lung and pulmonary arterial tree segmentation process resulting in measurement differences between observers. Linear regression and intraclass correlation coefficients (ICC) were used to compare these differences. In addition, author N.L. performed a blinded intraobserver analysis after more than a 6-month period from the initial measurements. The intraobserver analysis only included the first acquisition for each pair of helical scans for the 16 animals, with ICC, as well as linear regression plots used to assess agreement. For both inter- and intraobserver analyses, the mean ± standard deviation was reported for average absolute and percent differences.

Intraclass correlation coefficients and their 95% CIs were calculated using SPSS version 22 (SPSS Inc, Chicago, IL). The ICC was based on a single measure, absolute agreement, two-way random effects model. ICC values were interpreted using the following guidelines from Koo and Li [15]: less than 0.50 is poor reliability, between 0.50 and 0.75 is moderate reliability, between 0.76 and 0.90 is good reliability, and greater than 0.90 is excellent reliability.

## Results

A total of 46 acquisitions were analyzed, comprising 23 global mass measurement comparisons. For regional analysis, 138 comparisons were made between lobar segments, generating 276 segments across all animals. The average CT dose index for a single helical scan was 1.14 mGy.

### Qualitative analysis

**Fig. 2:**
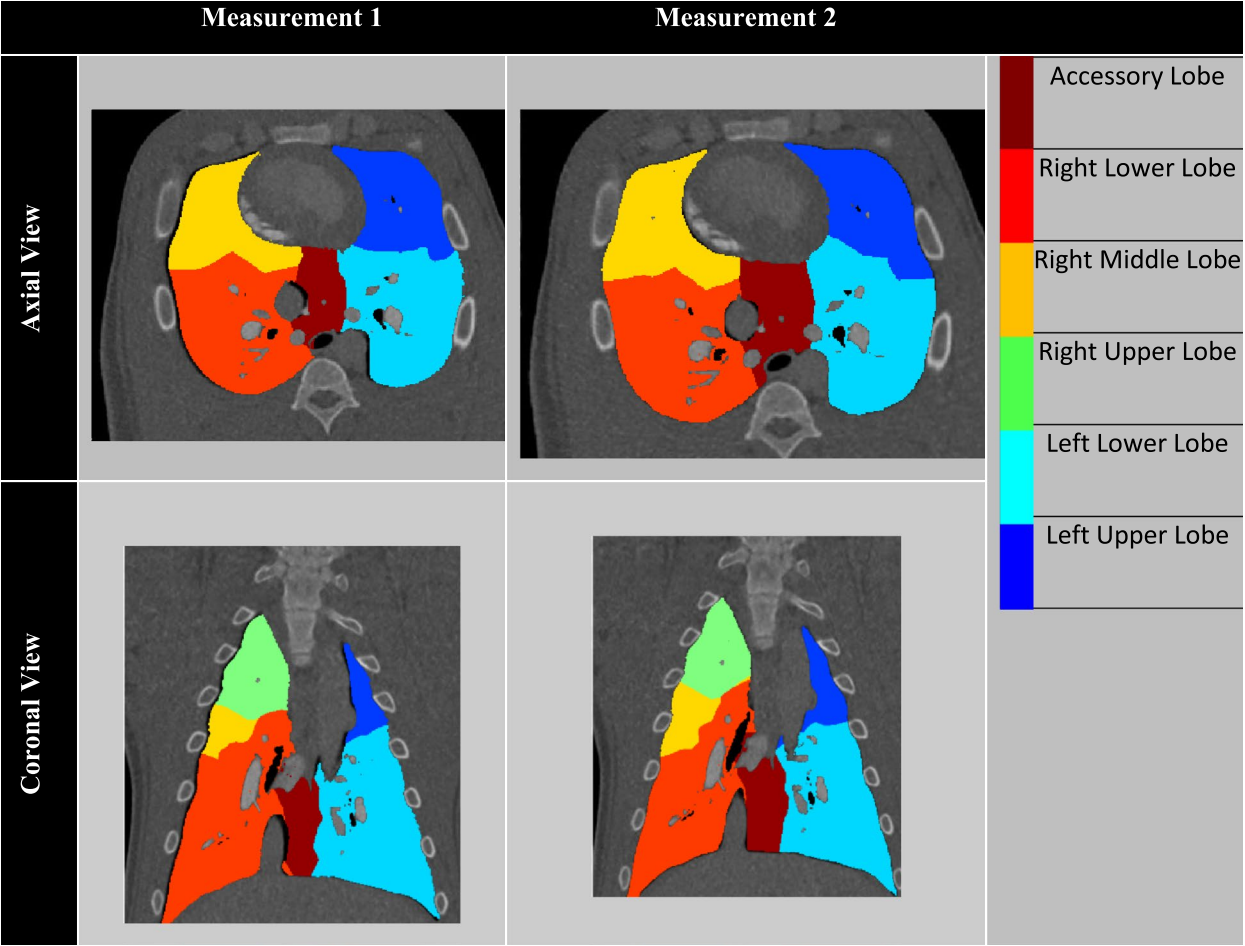
Qualitative representation of assignment maps. Two lobar assignments were conducted on two consecutive helical acquisitions using the minimum cost path − MCP technique. The axial and coronal views of the lobar segmentation are shown

Figure 2 illustrates the assignment territories generated using the MCP technique, showcasing both coronal and axial views. Different colors indicate the accessory lobe (AL), right lower lobe (RLL), right middle lobe (RML), right upper lobe (RUL), left lower lobe (LLL), and left upper lobe (LUL).

### Quantitative analysis

#### Global measurements

**Fig. 3:**
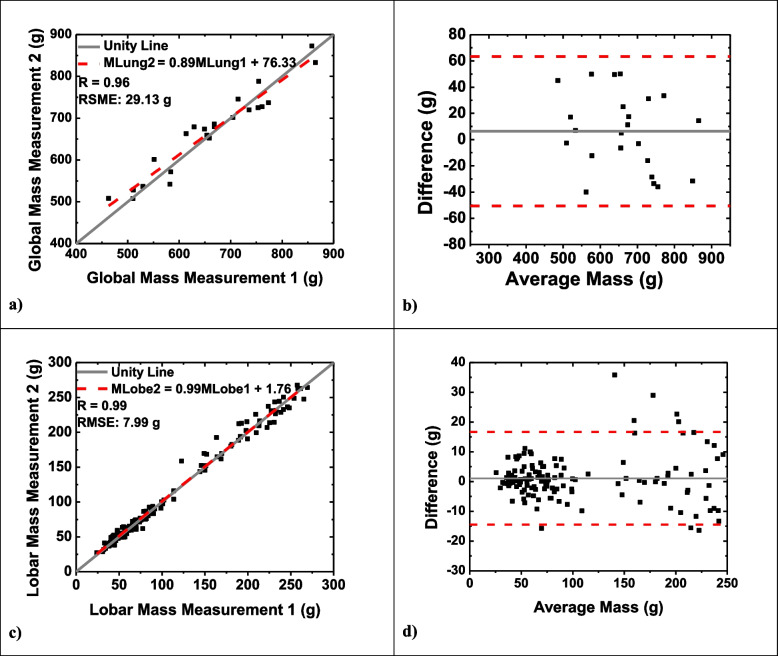
Quantitative analysis of MCP mass measurements. Linear regression analysis comparing the global (**a**) and lobar (**c**) mass measurement to a repeated computed tomography mass measurement. The dotted red line represents the regression line, with global mass correlated by MLung2 = 0.89MLung1 + 76.33 (*r* = 0.96; *p* = 0.306; RMSE = 29.13 g) and lobar mass correlated by MLobe2 = 0.99MLobe1 + 1.76 (*r* = 0.99; *p* = 0.120; RMSE = 7.99 g). Bland–Altman analysis was performed for the global (**b**) and lobar (**d**) mass measurement. Dotted red lines represent upper and lower bounds of agreement at mean ± 1.96 standard deviation, while the solid gray line represents the average difference. *MCP* Minimum cost path, *RMSE* Root mean square error

The average lung mass for the global measurements was 663.7 ± 103.7 g. The mass of the lungs for one acquisition was compared to the mass of a subsequent acquisition as detailed in Table 1. The global regression analysis for mass is shown in Fig. 3a, with specific regression details in Table 3. Performing a paired *t*-test between the two measurement conditions resulted in a *p* value of 0.306. The RMSE was found to be 29.1 g. Normalizing the RMSE to the mean lung mass resulted in a normalized RMSE of 4.4% suggesting low variance between the measurements. For the Bland–Altman plot illustrated in Fig. 3b, an average difference of 6.35 g (95% CI: -50.62, 63.32) was found. Additionally, an average percent difference between global mass measurements was 3.80 ± 2.69%. The calculated whole lung mass measurements from the first (MLung1) and second (MLung2) CT acquisitions were correlated by MLung1 = 0.89 MLung2 + 76.33 g (*r* = 0.96).

**Table 1:** Quantitative CT lung mass measurements

	Number of pairs	Average mass (g)	Average mass measurement 1 (g)	Average mass measurement 2 (g)	*p*-value	RMSE (g)	Normalized RMSE (%)
Global							
Overall	23	663.7 ± 103.7	660.5 ± 108.7	666.9 ± 100.7	0.306	29.1	4.4
Lobar							
Overall	138	110.6 ± 73.6	110.1 ± 73.8	111.2 ± 73.7	0.120	8.0	7.2
LUL	23	80.9 ± 17.2	81.4 ± 17.4	80.3 ± 17.3	0.362	5.5	6.8
LLL	23	198.7 ± 37.8	197.1 ± 40.7	200.3 ± 35.5	0.263	13.3	6.7
RUL	23	71.2 ± 13.1	71.4 ± 13.1	71.0 ± 13.3	0.659	4.0	5.6
RML	23	54.9 ± 12.5	54.2 ± 12.9	55.6 ± 12.3	0.132	4.6	8.4
RLL	23	216.5 ± 32.8	216.0 ± 34.1	217.1 ± 32.2	0.627	10.8	5.0
AL	23	41.5 ± 8.2	40.5 ± 7.3	42.6 ± 9.0	0.040	4.9	11.7

Data are expressed as mean ± standard deviation

*AL *Accessory lobe, *CT* Computed tomography, *LUL L*eft upper lobe, *LLL *Left lower lobe, *RUL *Right upper lobe, *RML *Right middle lobe, *RLL *Right lower lobe, *RMSE *Root mean square error

**Fig. 4:**
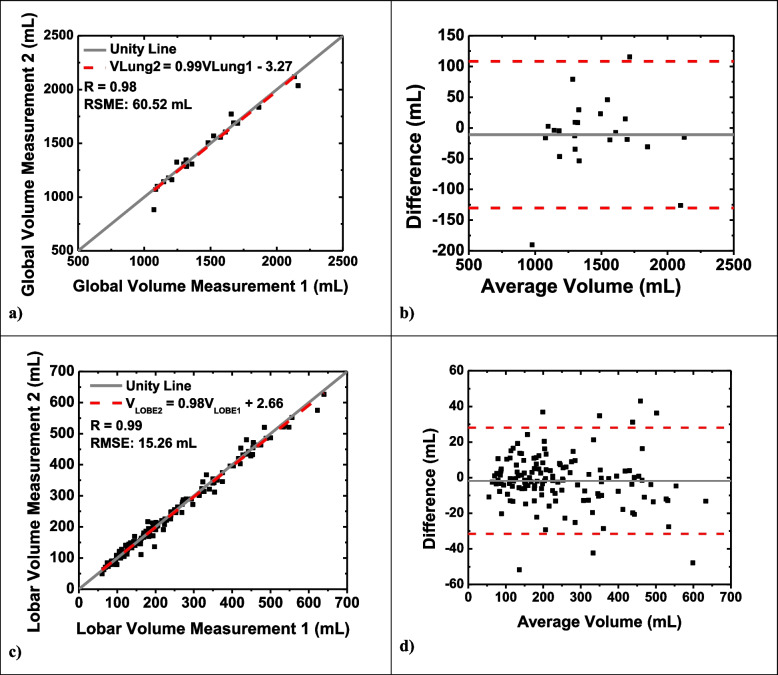
Quantitative analysis of MCP volume measurements. Linear regression analysis comparing the global (**a**) and lobar (**c**) volume measurement to a repeated computed tomography volume measurement. The dotted red line represents the regression line, with global volume correlated by VLung2 = 0.99 VLung1 − 3.27 (*r* = 0.98; *p* = 0.396, RMSE = 60.52 mL) and lobar mass correlated by VLobe2 = 0.98 VLobe1 + 2.66 (*r* = 0.99; *p* = 0.160; RMSE = 15.26 mL). Bland–Altman analysis was performed for the global (**b**) and lobar (**d**) mass measurement. Dotted red lines represent upper and lower bounds of agreement at mean ± 1.96 standard deviation, while the solid gray line represents the average difference. *MCP* Minimum cost path, *RMSE* Root mean square error

When analyzing the volume measurements, an average volume of 1,444.0 ± 309.1 mL was found. Similar to the mass calculations, volume for one acquisition was compared exclusively with a subsequent acquisition. Specific details can be seen in Table 2. Figure 4a, b demonstrates the global regression analysis and Bland–Altman plots respectively, with additional measurements detailed in Table 3. A *p-*value of 0.396 was found for the difference in volume measurements after performing a paired *t*-test between the two measurement conditions. The RMSE for the global volume measurement was 60.52 mL, and when normalizing this to the mean, a 4.2% normalized RMSE was found. For the Bland–Altman plot, an average difference of -10.98 mL (95% CI: -130.26, 108.29) was calculated. An average percent difference of 2.89 ± 4.11% was found for volume measurements. The calculated whole lung volume measurements from the first (VLung1) and second (VLung2) CT acquisitions were correlated by VLung1 = 0.99 VLung2 − 3.27 (*r* = 0.98).

**Table 2:** Quantitative CT lung volume measurements

	Number of pairs	Average volume (mL)	Average volume measurement 1 (mL)	Average volume measurement 2 (mL)	*p*-value	RMSE (mL)	Normalized RMSE (%)
Global							
Overall	23	1444 ± 309.1	1450 ± 310.4	1439 ± 314.7	0.396	60.5	4.2
Lobar							
Overall	138	240.7 ± 134.1	241.6 ± 135.2	239.8 ± 133.5	0.160	15.2	6.3
LUL	23	211.1 ± 71.2	212.2 ± 71.1	209.9 ± 72.9	0.233	9.0	4.2
LLL	23	378.3 ± 122.4	379.1 ± 127.3	377.6 ± 120.1	0.716	19.6	5.2
RUL	23	185.6 ± 36.5	188.0 ± 36.1	183.28 ± 37.6	0.160	15.8	8.5
RML	23	161.7 ± 45.8	161.2 ± 46.5	162.2 ± 46.0	0.773	16.4	10.2
RLL	23	408.9 ± 80.5	411.2 ± 80.0	406.5 ± 82.7	0.245	18.9	4.6
AL	23	98.7 ± 25.6	98.1 ± 24.7	99.2 ± 27.1	0.484	7.6	7.7

Data are expressed as mean ± standard deviation

*AL *Accessory lobe, *CT *Computed tomography *LUL *Left upper lobe, *LLL *Left lower lobe, *RUL *Right upper lobe, *RML *Right middle lobe, *RLL *Right lower lobe, *RMSE *Root mean square error

**Table 3:** Linear regression analysis for mass and volume measurements

Mass						Volume				
	Slope	Intercept	Pearson r	CCC (95% CI)	Average percent difference (mean ± SD)	Slope	Intercept	Pearson *r*	CCC (95% CI)	Average percent difference (mean ± SD)
*Global*						*Global*				
Overall	0.89	76.32	0.96	0.96 (0.91, 0.98)	3.80 ± 2.69	0.99	-3.27	0.98	0.98 (0.96, 0.99)	2.89 ± 4.11
*Lobar*						*Lobar*				
Overall	0.99	1.76	0.99	0.99(0.99, 1.00)	5.58 ± 5.39	1.01	2.66	0.99	0.99 (0.99, 1.00)	5.13 ± 5.88
LUL	0.95	3.16	0.95	0.95 (0.93, 0.96)	5.52 ± 4.78	1.02	-5.90	0.99	0.99 (0.99, 0.99)	4.59 ± 5.03
LLL	0.83	36.78	0.94	0.94 (0.91, 0.95)	5.02 ± 6.06	0.93	23.76	0.99	0.99 (0.98, 0.99)	4.05 ± 4.11
RUL	0.97	2.01	0.95	0.95 (0.90, 0.98)	4.27 ± 3.73	0.95	4.50	0.91	0.91 (0.80, 0.96)	5.20 ± 7.47
RML	0.89	7.21	0.93	0.93 (0.85, 0.97)	6.25 ± 5.86	0.93	12.99	0.93	0.93 (0.86, 0.97)	7.06 ± 8.33
RLL	0.89	23.93	0.94	0.94 (0.88, 0.98)	4.14 ± 3.22	1.01	-7.37	0.97	0.97 (0.94, 0.99)	3.57 ± 3.53
AL	1.07	-0.76	0.82	0.82 (0.64, 0.92)	8.27 ± 7.08	1.05	-4.15	0.96	0.96 (0.90, 0.98)	6.30 ± 5.11

*AL *Accessory lobe, *CCC *Concordance correlation coefficient, *CI *Confidence intervals, *LUL *Left upper lobe, *LLL *Left lower lobe, *RUL *Right upper lobe, *RML *Right middle lobe, *RLL *Right lower lobe, *SD *Standard deviation

#### Lobar measurements

Table 1 details the average mass for each lobe, along with the average mass for measurements 1 and 2 obtained from the MCP technique. Linear regression analysis for lobar mass measurement is presented in Fig. 3c, with specific details in Table 3. When comparing all lobes, a *p*-value of 0.120 was found. For the LUL, LLL, RUL, RML, and RLL, the *p*-values were all ≥ 0.132, while for the AL the *p*-value was 0.040. The RMSE was found to be 5.39 g, with a normalized RMSE equal to 7.2%. Bland–Altman analysis plots are shown in Fig. 3d, revealing an average difference of 1.06 g (95% CI: -14.53, 16.64). An average percent difference of 5.58 ± 5.39% was found for all lobar mass measurements. The lobar mass measurements from the first (MLobe1) and second (MLobe2) CT acquisitions were correlated by MLobe1 = 0.99MLobe2 + 1.76 (*r* = 0.99).

For lobar volume analysis (Table 2), Fig. 4c displays the linear regression analysis. The lobar volume measurements from the first (VLobe1) and second (VLobe2) CT acquisitions were correlated by VLobe1 = 0.98VLobe2 + 2.66 (*r* = 0.99) with a *p*-value of 0.160. For all lobes, we found a *p*-value for the volume measurements ≥ 0.160. The RMSE was found to be 15.26. Normalizing this to the mean resulted in a normalized RMSE of 6.3%. The Bland–Altman plot for the lobar volume measurement is provided in Fig. 4d, showing an average difference of -1.83 mL (95% CI: -31.64, 27.98). Additionally, an average percent difference of 5.12 ± 5.88% was found for lobar volume measurements.

### Intraobserver and interobserver variability

**Fig. 5:**
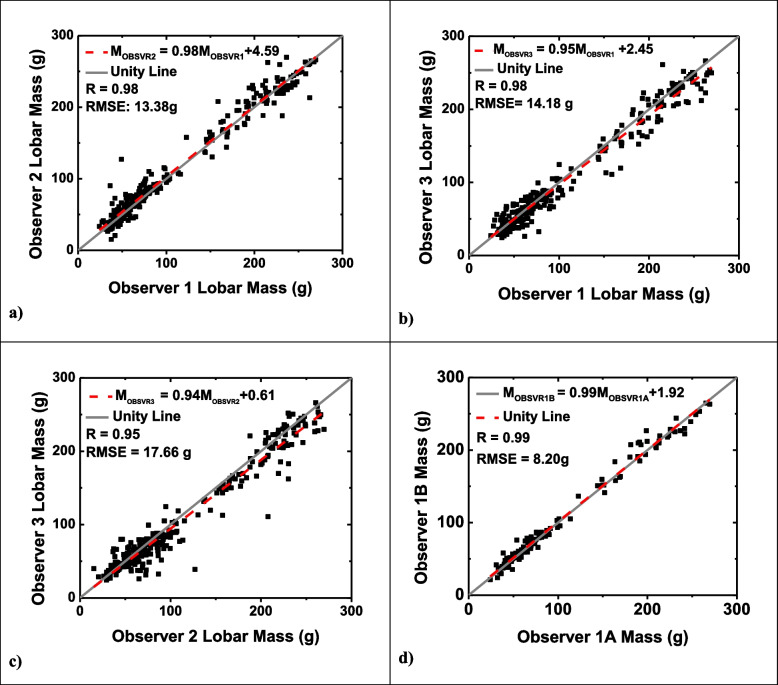
Linear regression plots for interobserver and intraobserver mass analysis. Linear regression analysis comparing observer 1 mass measurement to observer 2 mass measurement (**a**), observer 1 to observer 3 mass measurement (**b**), observer 2 to observer 3 mass measurement (**c**), and observer 1 initial mass measurement to observer 1 repeated mass measurement six months later (**d**). The dotted red line represents the regression line. *RMSE* Root mean square error

**Fig. 6:**
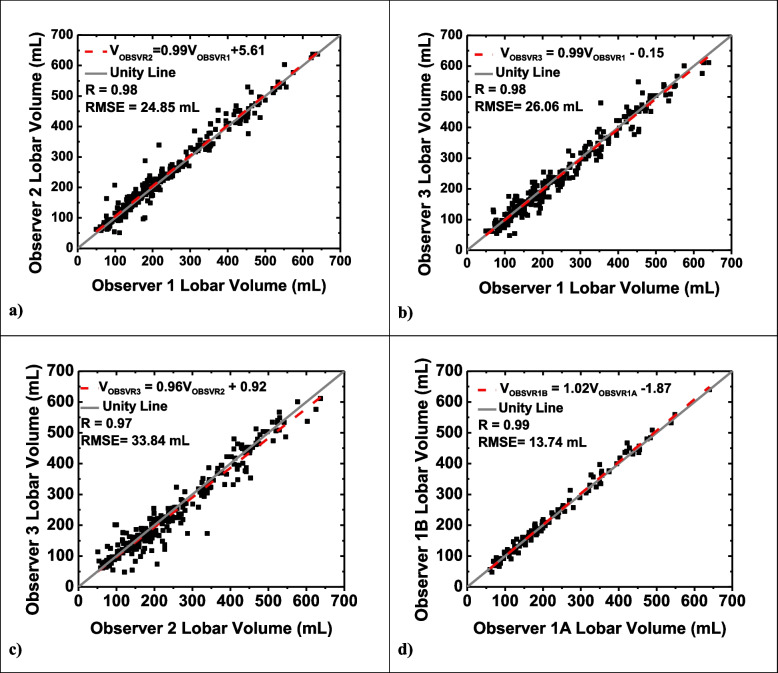
Linear regression plots for interobserver and intraobserver volume analysis. Linear regression analysis comparing observer 1 volume measurement to observer 2 volume measurement (**a**), observer 1 to observer 3 volume measurement (**b**), observer 2 to observer 3 volume measurement (**c**), and observer 1 initial volume measurement to observer 1 repeated volume measurement six months later (**d**). The dotted red line represents the regression line. *RMSE* Root mean square error

For the intraobserver lobar variability, 120 comparisons were made. The average absolute lobar mass difference between the two measurements was 6.03 g (RMSE = 8.20 g; *r* = 0.99). The average absolute lobar volume difference was 10.33 mL (RMSE = 13.74 mL, *r* = 0.99). Linear regression plots for mass and volume assessments are shown in Figs. 5 and 6. The ICC for both mass and volume measurements between the two measurements was 0.99 (excellent reliability), as reported in Table 4.

**Table 4:** Overall agreement lobar analysis

Mass				Volume		
	ICC (95% CI)	Average absolute difference (g) (mean ± SD)	Average percent difference (mean ± SD)	ICC (95% CI)	Average absolute difference (mL) (mean ± SD)	Average percent difference (mean ± SD)
Between all observers	0.978 (0.972, 0.983)	−	−	0.978 (0.972, 0.982)	−	−
Observer 1 *versus* 1	0.993 (0.991, 0.995)	6.03 ± 5.58	7.12 ± 7.22	0.994 (0.992, 0.996)	10.33 ± 9.10	5.68 ± 5.71
Observer 1 *versus* 2	0.983 (0.978, 0.987)	8.81 ± 10.08	10.19 ± 12.71	0.983 (0.978, 0.987)	16.40 ± 18.71	8.46 ± 11.01
Observer 1 *versus* 3	0.981 (0.974, 0.985)	10.25 ± 9.82	12.24 ± 12.89	0.981 (0.976, 0.985)	19.02 ± 17.84	9.80 ± 11.21
Observer 2 *versus* 3	0.970 (0.953, 0.980)	12.03 ± 12.95	13.95 ± 16.41	0.969 (0.959, 0.976)	23.23 ± 24.64	12.09 ± 15.51

*ICC *Intraclass correlation coefficient, using single measures, absolute agreement, two-way random effects model

For the interobserver lobar analysis, 276 comparisons were made between two observers. The average absolute lobar mass difference between observer 1 (N.L.) and observer 2 (N.V.) was 8.81 g (RMSE = 13.38 g; *r* = 0.98), while the absolute difference between observer 1 and observer 3 (A.Z.) was 10.25 g (RMSE = 14.18 g; *r* = 0.98). An average absolute difference of 12.03 g was found between observer 2 and observer 3 (RMSE = 17.66 g; *r* = 0.95).

In terms of volume measurements, the average absolute lobar volume difference between observer 1 and observer 2 was 16.04 mL (RMSE = 24.85 mL; *r* = 0.98). Observer 1 and observer 3 differed in the volume measurements by 19.02 mL on average (RMSE = 26.06 mL; *r* = 0.98). The average absolute difference between observer 2 and observer 3 was found to be 23.23 (RMSE = 33.84 mL; *r* = 0.97). Further details are shown in Table 4. Linear regression plots between the different observers for mass and volume assessments are shown in Figs. 5 and 6, respectively.

The ICC for both mass and volume measurements between observer 1 and observer 2, observer 1 and observer 3, and observer 2 and observer 3 was 0.98, 0.98, and 0.97, respectively (excellent reliability). When testing mass and volume measurements between all three observers, excellent reliability was found, as the ICC for both mass and volume measurements was 0.98. The overall results are detailed in Table 4.

## Discussion

Our study underscores the excellent reliability achieved by the MCP technique in lobar mass and volume measurements from noncontrast CT acquisitions of the lungs. Statistical analyses revealed no significant differences between two noncontrast CT scans for both global and lobar mass and volume measurements. Notably, our findings demonstrated no significant differences in volume measurements for the accessory lobe, but a significant discrepancy was identified in mass measurements for this lobe. The results of quantitative lobar mass and volume measurements indicate that the automated MCP technique can potentially provide clinically consistent lobar segmentation.

Regarding interobserver and intraobserver variability, the ICCs showed excellent agreement between all observers, indicating consistent and reliable measurements using the MCP technique between multiple observers. Overall, these assessments underscore the feasibility and potential clinical utility of employing the MCP technique for lobar segmentation on noncontrast CT scans.

Various techniques have been previously reported for lung lobe segmentation, each with its set of strengths and limitations. For example, reproducibility in lobar segmentation was assessed in single photon emission computed tomography–SPECT/CT images of lung cancer patients using software relying on fissure lines [3]. Although this software exhibited excellent reproducibility, its reliance on fissure lines poses challenges, especially in cases of incomplete fissure lines or unclear boundaries. These challenges can impact the precision and reliability of the segmentation process. Alternative strategies, such as one relying on zonal regions of interest without explicit anatomical significance, have also been proposed for lung lobe delineation [10]. However, this method lacks strong anatomical grounding, potentially compromising segmentation precision.

In contrast, the proposed MCP technique offers a distinctive approach by leveraging the closest vascular structure, particularly the lobar arterial subtree, to assign each tissue voxel for lobar segmentation. This approach is likely to provide advantages in terms of accuracy and reproducibility compared to other methodologies. Furthermore, the field has seen an increased adoption of machine learning and deep learning methodologies for lobar information assessment. This demonstrates the evolving landscape of methodologies employed for lung lobe segmentation, with an increasing emphasis on advanced computational approaches.

The MCP technique, with its reliance on vascular structures for segmentation, stands out as a promising alternative that combines anatomical precision with computational efficiency. As demonstrated in this study, the MCP technique shows excellent reproducibility in lobar mass and volume measurements from noncontrast CT images. This underscores its potential as a reliable and clinically applicable method for lung lobe segmentation, offering advantages over traditional approaches relying on fissure lines or less anatomically grounded strategies.

The MCP technique has undergone previous validation using CT pulmonary angiography images, where a comparative analysis between MCP-derived territories and dynamic CT perfusion-derived territories was conducted [11]. The results from this validation study revealed a mean Dice similarity coefficient of 0.84 ± 0.08 (mean ± standard deviation) for all tested conditions. This coefficient, assessing the spatial overlap between segmented regions, signifies that the MCP technique demonstrates precise spatial correspondence. Furthermore, the validation process also addressed mass correspondence, providing additional confirmation of the accuracy of the MCP technique in assessments related to mass [11]. These findings support the reliability and validity of the MCP technique, reinforcing its utility in accurately delineating pulmonary arterial territories and associated mass characteristics.

The results of our current study demonstrate that the MCP technique is not only highly reproducible but can also be effectively applied using noncontrast CT for lobar segmentation. While certain studies have achieved success in utilizing contrast-enhanced CT images for lobar segmentations [1, 16], the availability of such images may be limited for some patients. Contrast-enhanced images offer improved visibility of smaller vessels, enabling a more precise extraction of the pulmonary arterial tree and lobar segmentation, thereby providing a more comprehensive depiction of the pulmonary tree. However, as illustrated by our study, the MCP technique is adaptable to noncontrast images. Through the application of the MCP technique with noncontrast CT images, specific treatment planning becomes feasible for patients who do not have access to contrast CT images. This adaptability enhances the applicability of the MCP technique in clinical assessments, broadening its utility to a wider range of patients.

Moreover, given the adaptability of the MCP technique between noncontrast and contrast images, it holds the potential to generate lobar-specific perfusion maps and perfusion defect maps using whole lung perfusion measurements [14]. Furthermore, the MCP technique has been successfully applied in CT pulmonary angiography to assist in the quantification of lung tissue at-risk distal to a pulmonary embolism [11]. This application enables the calculation of the total mass percentage at risk for the entire lung and specific lobes, thereby enhancing risk stratification for pulmonary embolism. Additionally, the regional analysis of lung mass and volume, along with the automatic segmentation of pulmonary vessels, is particularly crucial in patients with chronic obstructive pulmonary disease—COPD [17]. By identifying significant mass/volume changes, the MCP technique has the potential to facilitate the detection of pulmonary abnormalities, thus contributing to the development of appropriate treatment strategies [18].

This study has certain limitations that warrant consideration. First, a semiautomatic technique was employed to segment the pulmonary arterial tree, introducing the potential for human error. Although interobserver analyses demonstrated good agreement between observers, future studies should explore automated techniques to ensure consistent lung tissue segmentation and pulmonary arterial tree extraction. Second, most lung scans were conducted in relatively healthy states; however, some studies presented challenges such as fluid buildup, aspiration during intubation, heart failure due to high-volume injections, and iatrogenic trauma during intubation. These factors may have contributed to difficulties in extracting pulmonary arterial tree and lung tissue boundaries, particularly evident in the significant difference found in the accessory lobe for mass calculations. Third, the segmentation of small pulmonary arterial branches posed challenges, and artifacts or diseases in the distal portions of the arterial tree could introduce variation. Future studies may mitigate this by employing a fully automated method for vessel centerline extraction. Fourth, the reproducibility assessment was conducted in swine under well-defined breath-hold conditions with endotracheal intubation. For patients without reproducible breath-holds, volume changes between inspiratory and expiratory phases may affect volume measurements. However, mass theoretically should not change between these phases and therefore should not affect the reproducibility assessment in these patients. Fifth, the absence of tube modulation in our acquisitions may have implications for radiation dose and noise. Future studies could explore the use of tube modulation to further reduce these factors. Finally, the study was restricted to a single scanner, and while comparable results are anticipated, it is crucial to test the reproducibility of the MCP technique on other scanners to ensure generalizability.

In conclusion, the MCP technique exhibits excellent reproducibility in paired measurements on noncontrast CT scans in swine. This finding suggests the feasibility of employing this technique for lobar segmentation to be tested on humans.

## Data Availability

Data generated or analyzed during the study are available from the corresponding author by request.

## Abbreviations

AL: Accessory lobe
CI: Confidence interval
CT: Computed tomography
ICC: Intraclass correlation coefficient
LLL: Left lower lobe
LUL: Left upper lobe
MCP: Minimum cost path
RLL: Right lower lobe
RML: Right middle lobe
RMSE: Root mean square error
RUL: Right upper lobe
